# Deficiency of fibroblast activation protein alpha ameliorates cartilage destruction in inflammatory destructive arthritis

**DOI:** 10.1186/s13075-015-0524-6

**Published:** 2015-01-20

**Authors:** Stefan Wäldele, Christina Koers-Wunrau, Denise Beckmann, Adelheid Korb-Pap, Corinna Wehmeyer, Thomas Pap, Berno Dankbar

**Affiliations:** Institute of Experimental Musculoskeletal Medicine, University Hospital Muenster, Albert-Schweitzer-Campus 1, Bldg. D3, Muenster, D-48149 Germany

## Abstract

**Introduction:**

Inflammatory destructive arthritis, like rheumatoid arthritis (RA), is characterized by invasion of synovial fibroblasts (SF) into the articular cartilage and erosion of the underlying bone, leading to progressive joint destruction. Because fibroblast activation protein alpha (FAP) has been associated with cell migration and cell invasiveness, we studied the function of FAP in joint destruction in RA.

**Methods:**

Expression of FAP in synovial tissues and fibroblasts from patients with osteoarthritis (OA) and RA as well as from wild-type and arthritic mice was evaluated by immunohistochemistry, fluorescence microscopy and polymerase chain reaction (PCR). Fibroblast adhesion and migration capacity was assessed using cartilage attachment assays and wound-healing assays, respectively. For *in vivo* studies, FAP-deficient mice were crossed into the human tumor necrosis factor transgenic mice (hTNFtg), which develop a chronic inflammatory arthritis. Beside clinical assessment, inflammation, cartilage damage, and bone erosion were evaluated by histomorphometric analyses.

**Results:**

RA synovial tissues demonstrated high expression of FAP whereas in OA samples only marginal expression was detectable. Consistently, a higher expression was detected in arthritis SF compared to non-arthritis OA SF *in vitro*. FAP-deficiency in hTNFtg mice led to less cartilage degradation despite unaltered inflammation and bone erosion. Accordingly, FAP^−/−^ hTNFtg SF demonstrated a lower cartilage adhesion capacity compared to hTNFtg SF *in vitro*.

**Conclusions:**

These data point to a so far unknown role of FAP in the attachment of SF to cartilage, promoting proteoglycan loss and subsequently cartilage degradation in chronic inflammatory arthritis.

## Introduction

Rheumatoid arthritis (RA) is an autoimmune disease that primarily affects the joints and that is characterized by chronic inflammation and progressive cartilage and bone destruction [1]. Synovial inflammation and hyperplasia as well as invasion of synovial fibroblasts (SF) into the articular cartilage and erosion of the underlying bone, all are hallmarks of RA [2]. The major classes of proteolytic enzymes implicated in the degradation of cartilage are metalloproteinases (matrixmetalloproteinase (MMP), a disintegrin and metalloproteinase with thrombospondin motifs (ADAMTS)) and serine proteases [3,4]. Among the latter, fibroblast activation protein alpha (FAP) is a transmembrane serine protease with dual proteolytic activity, a dipeptidyl peptidase activity and a gelantinolytic activity [4]. Moreover, FAP is associated with cell migration and cell invasiveness [5,6] and has been linked to several diseases, including cancer and arthritis [6-9]. FAP is expressed in the rheumatoid synovium [9] and has been localized at the invadopodia of migrating lung fibroblasts [5]. Moreover, increased formation of invadopodia by synovial fibroblasts (SF) has recently been demonstrated to promote cartilage breakdown in a collagen-induced arthritis mouse model [10]. Although the proteolytic activity of FAP has been clearly linked to migration and invasion, the role of FAP in inflammatory matrix degradation remains controversial, since inhibition of both the proteolytic activity of FAP and dipeptidylpeptidase 4 (DPP4) showed increased cartilage invasion by rheumatoid arthritis SF [11]. In the present study, we therefore studied the *in vivo* role of FAP in joint destruction in a human tumor necrosis factor (TNF) transgenic (hTNFtg) mouse model of RA, characterized by progressive cartilage and bone loss [12].

## Methods

### Mice

To assess the impact of FAP deficiency on TNF-mediated joint destruction, fap^−/−^ (FAP knockout) hTNFtg animals were generated by crossing hTNFtg mice (Tg197 strain; C57BL/6), which develop an inflammatory destructive arthritis animals [12] with fap^−/−^ mice (C57BL/6). Clinical signs of arthritis were determined once weekly as described previously [13]. The local ethics committee ‘Landesamt für Natur, Umwelt und Verbraucherschutz Nordrhein-Westfalen (LANUV)’ approved all animal procedures.

### Histomorphometric analysis

Hind paws at week 12 were fixed overnight in 4.0% formalin and then decalcified in EDTA. Paraffin-embedded sections were stained with toluidine blue for assessment of synovial inflammation, cartilage degradation and subchondral bone erosion [13,14]. Number of osteoclasts was evaluated by tartrate-resistant acid phosphatase (TRAP) staining of embedded sections. All analyses were performed using a Zeiss Observer.Z1 and an image analysis system (Zeiss AxioVision 4.8. software; Carl Zeiss, Marburg, Germany).

### Human synovial tissues

Samples of synovial tissues from patients with RA or osteoarthritis (OA) (according to the 1987 revised American College of Rheumatology criteria for RA and OA) were obtained at joint replacement surgery. All patients gave written consent and the studies were approved by the ethics committees of the ‘Ärztekammer Westfalen-Lippe; and of the Medical Faculty of the Westfälische Wilhelm-University Muenster.

### Human and mouse fibroblasts

Human and mouse SF were isolated by enzymatic digestion of synovial tissues and hind paws, respectively. All cells were cultured in Dulbecco’s modified Eagle’s medium (DMEM) with 10% fetal calf serum (FCS) at 37°C and 5% CO_2_. Human and murine SF were used until passages 4.

### Immunohistochemical and immunofluorescence staining

For immunohistochemistry, paraffin-embedded sections were pretreated with Trypsin/EDTA for 15 min at 37°C, blocked with 5% horse serum and stained with rabbit anti-human FAPalpha antibodies (Abcam, Cambridge, MA, USA) or rabbit immunoglobulin G (IgG). Detection was performed with an alkaline phosphatase technique (Vectastain, Vector Laboratories, Burlingame, CA, USA). Methyl green was used for counterstaining (Vector Laboratories).

For immunofluorescence staining, human fibroblasts were fixed and permeabilized with 4% PFA, washed and quenched with 0.1 M NH_4_Cl. After blocking with 2% horse serum, cells were stained with anti-human FAPalpha antibodies and labeled by a secondary AlexaFluor488 antibody (Life Technologies, Darmstadt, Germany). Nuclei were stained using DAPI, the cytoskeleton using phalloidin.

### RT-PCR and genotyping

cDNAs were synthesized from total RNAs obtained from OASF and RASF by reverse-transcriptase. FAP was amplified by polymerase chain reaction (PCR) using *Taq* polymerase (Peqlab) and the following primer: human FAP forward GTTATTGCCTATTCCTATTATG; reverse GTCCATCATGAAGGGTGGAAA [9]; glyceraldehyde-3-phosphate dehydrogenase (GAPDH) served as control [15]. Genomic DNA was isolated from ear stamps and used for genotyping of FAP-deficient mouse strains using the REDExtract-N-Amp™ Tissue PCR Kit (Sigma-Aldrich, St Louis, MO, USA) and the following primer: FAP forward GGAAGACAAGGTGTATCTGTGG; reverse GTGTTTTCTGCTACTTGAGAATAATCGG; neomycin cassette forward GGCCATTGAACAAGATGGAT; reverse GTAGCCGGATCAAGCGTATG.

### *In vitro* migration assay

To assess migration of SF *in vitro*, a wound-healing assay was used (ibidi, Martinsried, Germany). 2.5 × 10^4^ SF were seeded in two cell culture reservoirs each, which were separated by a 500 μm thick wall. After removal of the silicone insert from the surface, closure of the created cell-free gap (wound) by cell migration was quantified.

### *In vitro* attachment assay

For attachment analyses, cartilage of the femoral head of 4- to 6-week-old wild-type (wt) mice was obtained aseptically. 6 × 10^4^ SF were seeded onto pretreated cartilage (1 ng/ml for 24 h) for 2 h under continuous rotation. Following further 12 h of incubation, cartilage and attached cells were fixed in 4.0% formalin, stained with hematoxylin and the numbers of attached SF were across the whole cartilage surface were counted [14].

### Statistical analysis

Data are presented as means ± standard error of the mean (SEM). Differences between two independent groups were analyzed by the Mann–Whitney test. A value of less than *P* <0.05 was considered statistically significant.

## Results

### FAP is highly expressed in RA synovial tissues and fibroblasts

**Figure 1:**
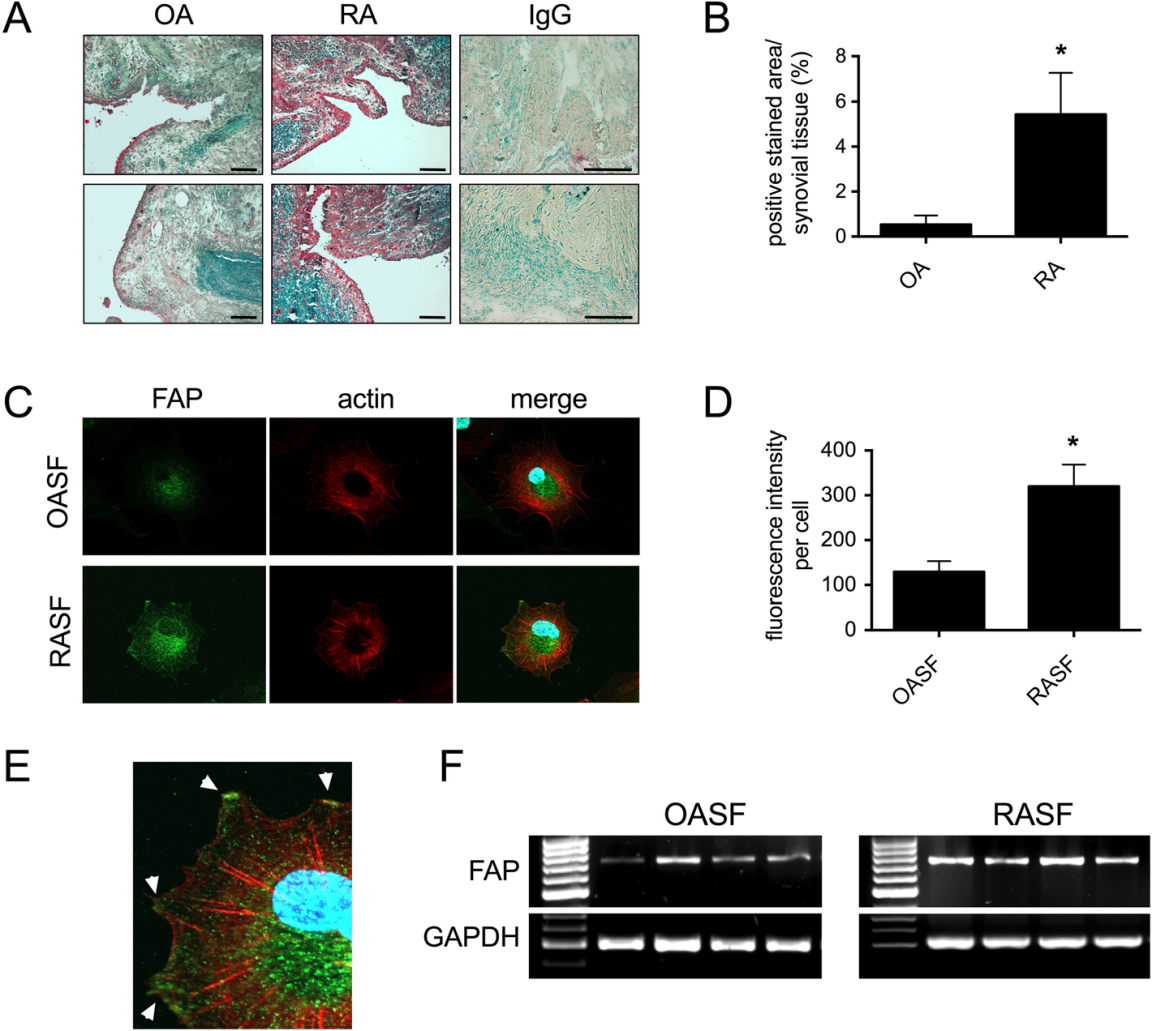
**Expression of fibroblast activation protein (FAP) in RA. (A)** Immunohistochemical staining of FAP (red) in synovial tissues of subjects with osteoarthritis (OA; n = 4) and rheumatoid arthritis (RA; n = 4). Counterstaining was methyl green. Scale bars, 200 μm. **(B)** Quantification of FAP positivity presented as percentage of positive stained area of synovial tissue. **(C)** Expression of FAP in OA synovial fibroblasts (OASF, n = 3) and RA synovial fibroblasts (RASF, n = 3) by immunofluorescence staining; green: FAP, red: actin cytoskeleton, blue: nucleus. **(D)** Quantification of fluorescence intensity per cell (n = 12, three independent patients). **(E)** Magnified section from (C) showing localization of FAP at membrane protrusions of RASF (white arrows). **(F)** Identification of human FAP mRNA transcripts in OASF and RASF by RT-PCR; #1-4 indicate independent patients. Glyceraldehyde-3-phosphate dehydrogenase (*GAPDH*) served as control.

To assess whether FAP may be involved in the pathology of RA, we first investigated whether a higher expression of FAP in synovial tissues as well as in SF of RA patients is evident. Indeed, we observed a high expression of FAP in RA throughout the whole synovial membranes. In contrast, only marginal expression, predominantly in the lining layer, was detectable in synovial tissues from OA patients that were used as non-inflamed controls (Figure 1A and B). In line with these data, SF obtained from RA patients demonstrated a higher expression of FAP transcripts compared to SF from OA patients (Figure 1F) and a higher expression was observed in RASF compared to OASF, as reflected by significantly higher fluorescence levels (Figure 1C and D). Moreover, FAP was localized to protrusive membrane structures of RASF (Figure 1E).

### FAP deficiency does not affect clinical symptoms of arthritis

**Figure 2:**
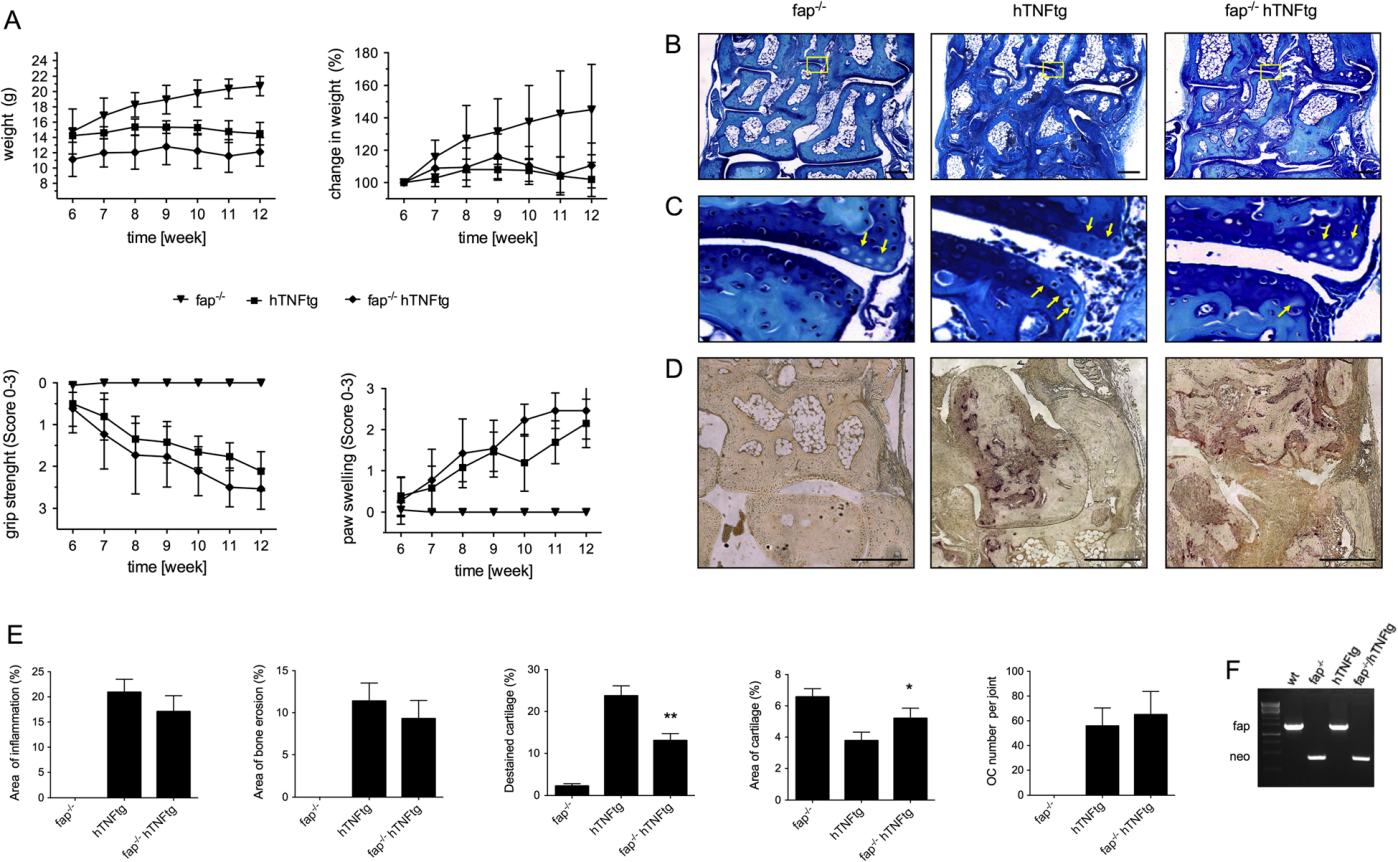
**Effect of fibroblast activation protein (FAP) deficiency on disease severity in arthritic mice. (A)** Effect of FAP-deficiency on weight, grip strength, and paw swelling over time (weeks 6 to 12). All data are means ± standard error of the mean (SEM) of 7 FAP knockout (fap^−/−^), 13 human tumor necrosis factor alpha transgene (hTNFtg) and 13 (fap^−/−^ hTNFtg) mice **(B)** Representative microphotographs of toluidine blue-stained joint sections at week 12 of fap^−/−^, hTNFtg, and fap^−/−^/ hTNFtg mice, illustrating inflammation and joint destruction. **(C)** Magnified representative cartilage areas, demonstrating destained cartilage caused by proteoglycan loss (arrows). **(D)** Tartrate-resistant acid phosphatase (TRAP) staining of corresponding joint sections, illustrating the presence of osteoclasts (red-brownish). Scale bars, 200 μm. **(E)** Quantitative histomorphometric assessment of synovial inflammation, bone erosion, cartilage degradation, and osteoclast numbers in tarsal joints. All data are means ± SEM of 7 fap^−/−^, hTNFtg, and fap^−/−^ hTNFtg mice each (^*^ *P* <0.05, ^**^ *P* <0.01). **(F)** Representative genotyping PCR for confirmation of FAP deficiency in the various mouse lines.

To investigate a possible role of FAP in the pathology of arthritis, FAP-deficient hTNFtg mice were compared with hTNFtg mice. As expected, hTNFtg mice spontaneously developed an inflammatory arthritis between week 6 and 12, which clinically presented as progressive increase in paw swelling and loss in grip strength and morphometrically by joint destruction (Figure 2). Surprisingly, FAP-deficiency in hTNFtg mice did not lead to significant reduction of signs of arthritis as compared to hTNFtg mice. In detail, body weight was lower in fap^−/−^ hTNFtg than in hTNFtg mice and did not gradually increase over time in both genotypes. Additionally, grip strength decreased and paw swelling increased during the progression of the disease, again displaying no differences between the two genotypes. No clinical signs of arthritis were observed in fap^−/−^ mice (Figure 2A).

### FAP deficiency ameliorates cartilage degradation *in vivo*

In order to investigate the impact of FAP on TNF-mediated joint destruction, we next analyzed whether the lack of FAP leads to altered histological changes in the arthritic joints of hTNFtg mice (Figure 2B). Histomorphometric analyses of hind paws demonstrated that fap^−/−^ hTNFtg mice displayed similar synovial inflammation and bone erosion compared to hTNFtg mice, which was accompanied by equal number of osteoclasts in the inflamed joints (Figure 2D and E). Interestingly, FAP deficiency ameliorated cartilage damage in the arthritic joints, as demonstrated by significantly increased cartilage area (about 30%) and proteoglycan content, displayed by a significant decrease in destained cartilage by about 45% (Figure 2C and E).

### FAP deficiency reduces SF adhesion but not migration

**Figure 3:**
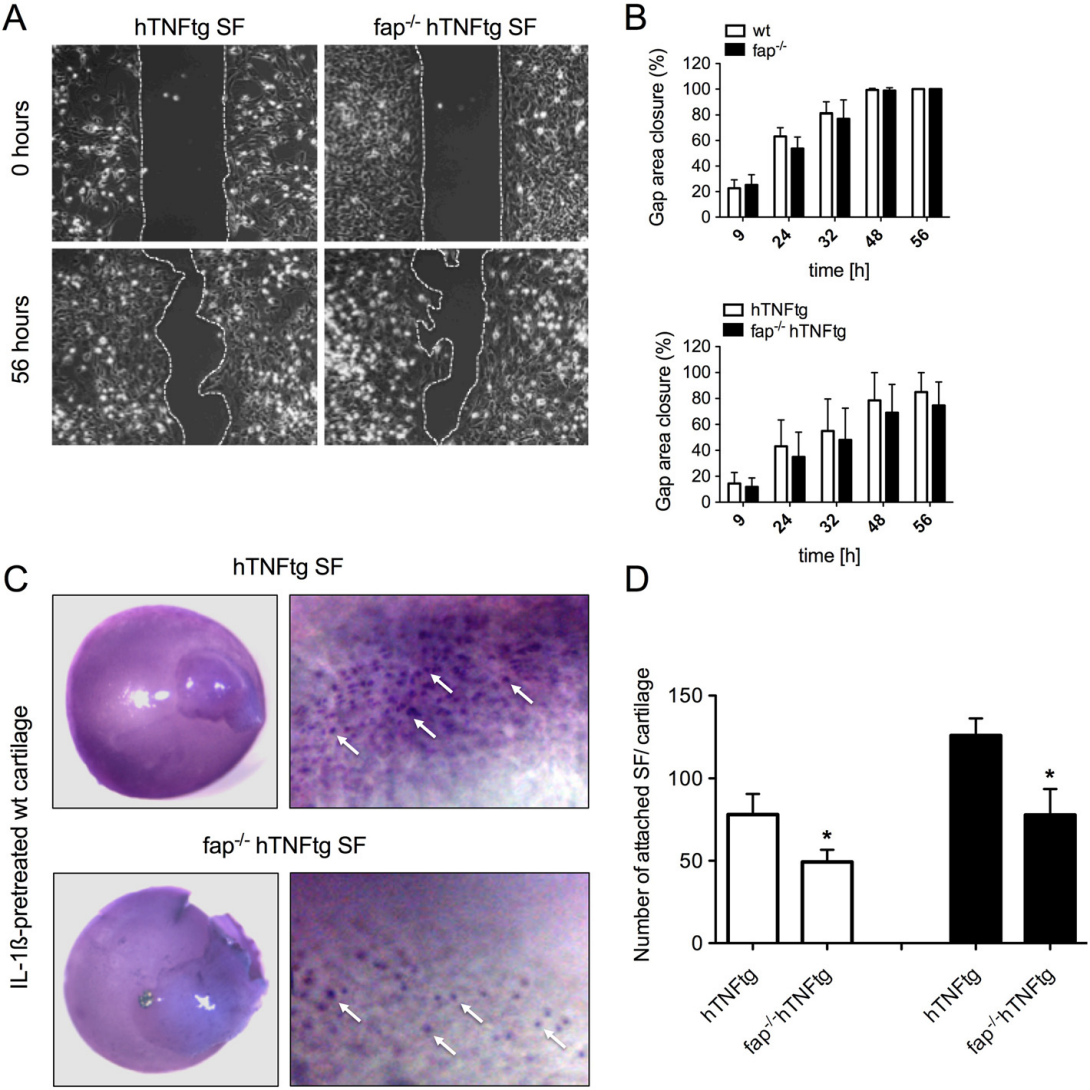
**Functional role of fibroblast activation protein (FAP) on synovial fibroblast behavior. (A)** Micophotographs of *in vitro* migration assays from human tumor necrosis factor alpha transgene (hTNFtg) and FAP knockout (fap^−/−^) hTNFtg SF demonstrating gap closure after 56 hours. **(B)** Quantification comparison of gap closure between wild-type (wt) and fap^−/−^ as well as hTNFtg and fap^−/−^ hTNFtg SF at indicated time points. All data are means ± standard error of the mean (SEM) of three mice each. **(C)** Representative pictures of *in vitro* attachment of hTNFtg and fap^−/−^ hTNFtg SF on wt cartilage, pretreated with 1 ng/ml interleukin 1 beta (IL-1β), after 14 hours. Cartilage and attached cells were stained with hematoxylin. **(D)** Quantitative assessment of attached synovial fibroblasts (SF) from hTNFtg and fap^−/−^ hTNFtg mice on both non-treated (white bars) and treated (black bars) wt cartilage. All data are means ± SEM of three mice each (^*^ *P* <0.05).

We next investigated the influence of FAP on fibroblast migration because SF have been implicated in inflammatory cartilage degradation (Figure 3A). Surprisingly, we observed no differences in the migration capacity of SF neither from wt and fap^−/−^ mice nor from hTNFtg and fap^−/−^ hTNFtg mice during the whole time course of 56 hours (Figure 3B). However, we found a significantly reduced attachment capacity of FAP-deficient SF to cartilage (Figure 3C). Fap^−/−^ hTNFtg SF demonstrated less adhesion of about 40% compared to hTNFtg SF, independent of whether the cartilage was pretreated with interleukin 1 beta ( IL-1ß) or not, indicating a role for FAP in SF adhesion to cartilage matrix (Figure 3D).

## Discussion

The progressive joint destruction in RA is considered to be mediated by aggressive, tumor-like SF that invade and destroy adjacent cartilage and bone. Migration and invasion of cells, particular tumor cells, rely on specialized structures called invadopodia, containing adhesion molecules and proteases responsible for extracellular matrix (ECM) degradation. Since the formation of invadopodia-like structures by arthritis SF has been associated with cartilage degradation [10] and additionally, FAP has been localized on invadopodia [5,6], we first investigated the expression of FAP in RA. A higher expression of FAP in the rheumatoid synovium as well as an increased expression by rheumatoid arthritis synovial fibroblasts (RASF) compared to OA led us to the hypothesis that increased FAP expression promotes matrix degradation in RA. To address this question, we investigated the influence of FAP-deficiency on joint destruction in the hTNFtg mouse model of RA. Analysis of the arthritic changes in these mice revealed that the lack of FAP had no effect on synovial hyperplasia and bone destruction in hTNFtg mice. Moreover, osteoclast numbers were equal in both genotypes, suggesting no involvement of FAP in inflammatory osteoclast development as well as bone erosion. Moreover, most strikingly, FAP deficiency broadly ameliorated cartilage degradation in hTNFtg mice, pointing to an important role of FAP in cartilage destruction in RA.

Although the proteolytic activity of FAP has been linked to migration, we did not observe any effects of FAP on the migratory activity of SF, pointing to a proteolytic-independent role of FAP in cartilage degradation. In this regard, attachment of synovial cells, particularly SF, to the cartilage matrix has shown to be an early event in the course of arthritis, contributing to matrix degradation [14]. Thus, our finding that adhesion of hTNFtg SF to wt cartilage was strongly reduced about 40% in the absence of FAP, strongly suggests that FAP has a considerable function in cell-matrix interaction and that this function is independent of its proteolytic activity.

Recently, Ospelt and colleagues demonstrated that inhibition of the dipeptidylpeptidase activity of both FAP and DPP-4 (DPP-4-like activity) led to increased cartilage invasion by rheumatoid arthritis SF. Interestingly, increased invasion appears to be mediated by elevated expression of MMPs through upregulated SDF-1 [11]. In addition to its dipeptidylpeptidase and gelatinolytic activity, FAP is known to interact with various cell surface molecules such as integrins and specific MMPs which interaction is not affected by inhibition of DPP4-like activity. In this context and of particular importance, the formation of a complex composed of integrin α3β1 and FAP on the cell surface has been demonstrated to be necessary for the formation of functional invadopodia on melanoma cells [16]. Moreover, this complex appears to be important not only for migration but also for adhesion, and that independently of the enzymatic activity of FAP [17]. In this context, a variety of integrins, especially those of the β1 family, have been found to be overexpressed in RASF [18,19] and it has been shown that blocking these integrins on the surface of RASF reduces their attachment and invasive capacity [20,21]. Thus, in the study by Ospelt and colleagues, the remaining adhesion capacity of the RASF together with the remaining gelatinase activity of FAP and the increased expression of MMPs probably contributed to the higher invasiveness of the RASF, whereas blocking the formation of integrin-FAP complexes on the surface of RASF in our study appears to account for decreased cartilage damage.

## Conclusions

Our study points to a so far unknown role of FAP in fibroblast-mediated cartilage degradation in arthritis. While the exact nature of the matrix molecules that are responsible for SF attachment remains to be determined, it appears for the first time that, in addition to integrins, FAP is involved prominently in fibroblast attachment and cartilage degradation. It is most likely that high expression of FAP and β1 integrins on arthritis SF leads to increased attachment of SF to cartilage matrix, thus promoting cartilage degradation.

Since the loss of articular cartilage is of particular importance because it is largely irreversible and thus constitutes a ‘point of no return’ in the destruction of RA joints, further understanding of the function of FAP may give rise to FAP as a potential target for countering cartilage degradation.

## Abbreviations

ADAMTS: a disintegrin and metalloproteinase with thrombospondin motifs
DMEM: Dulbecco’s modified Eagle’s medium
DPP4: dipeptidylpeptidase 4
ECM: extracellular matrix
FAP: fibroblast activation protein alpha
fap^−/−^: FAP knockout mouse
FCS: fetal calf serum
GAPDH: glyceraldehyde-3-phosphate dehydrogenase
hTNFtg: human tumor necrosis factor alpha transgene
IgG: immunoglobulin G
IL-1ß: interleukin 1ß
MMP: matrixmetalloproteinase
OA: osteoarthritis
OASF: osteoarthritis synovial fibroblasts
PCR: polymerase chain reaction
RA: rheumatoid arthritis
RASF: rheumatoid arthritis synovial fibroblasts
SF: synovial fibroblasts
TNF: tumor necrosis factor
TRAP: tartrate-resistant acid phosphatase
wt: wild-type
